# Surgical Management of Acute Abdomen in Adult Patients: Experience from a Private Hospital in Addis Ababa, Ethiopia

**DOI:** 10.4314/ejhs.v32i4.9

**Published:** 2022-07

**Authors:** Yonas Ademe, Nebyou Seyoum, Rakeb Lemma

**Affiliations:** 1 Addis Ababa University, College of Health Sciences, School of Medicine, Department of Surgery; 2 SUNY Upstate Medical University, School of Medicine, Syracuse, NY, USA

**Keywords:** Acute abdomen, acute appendicitis, surgery

## Abstract

**Background:**

Acute abdomen is a major surgical problem in Ethiopia with surgery for acute abdominal conditions accounting for roughly one-third of total emergency operations in many centers. This study was conducted with the aim of studying the pattern and outcome of surgically managed acute abdominal cases in a private general hospital in Addis Ababa, Ethiopia.

**Methods:**

This was a retrospective cross-sectional study of acute abdominal cases in adult patients operated at Teklehaimanot General Hospital between January 1, 2018 and August 1, 2019.

**Results:**

A total of 267 patients' medical records were reviewed. The male to female ratio was 1.5:1 and majority of patients were between the age range of 20 to 40 years with mean age of 36±16 years. The average duration of symptoms before arrival was 71.1±84.4 hours (range 3 to 504 hours) and only 85 (31.8%) of patients reached to the Hospital within 24 hours or less of onset of symptoms. Acute appendicitis was the most common cause of acute abdomen; observed in 193 (72.3%) of the cases. Overall post-operative complication rate was 14.8% and post-operative mortality rate was 1.9%. It was found that delayed presentation (OR 2.01, 95% CI 1.64–7.84), old age (OR 1.51, 95% CI 1.89–3.59), and tachycardia at presentation (OR 2.85, 95% CI 1.03–6.82) were major predictors of morbidity and mortality in operated patients.

**Conclusion:**

In this study acute appendicitis accounted for the majority of cases operated for acute abdomen. Length of post-operative hospital stay, early post-operative complication rate, and overall mortality rate were found to be significantly lower in our series than other reports.

## Introduction

Acute abdomen is defined as a sudden onset intra-abdominal disease which needs immediate surgical intervention (1). Acute abdominal pain can be caused by both surgical and non-surgical causes. Surgical causes include hemorrhage, infection, perforation, blockage, and ischemia in the abdominal cavity while non-surgical causes include toxins or drugs as well as endocrine, metabolic, or hematologic causes. Due to the presence of both surgical and non-surgical causes of acute abdominal pain, a thorough and quick work-up is required to identify the exact cause. Although imaging and laboratory studies for abdominal etiology have advanced, providers still heavily rely on history and physical examination for the accurate diagnosis of abdominal complaints.

Over the past two decades, several researchers have studied the prevalence, causes and post-operative outcomes of acute abdomen at different Ethiopian hospitals including Tikur Anbessa Specialized Hospital (Addis Ababa), Mekelle Hospital (Mekelle), Glen Olson Memorial Primary General Hospital (Butajira), Yirgalem Hospital (Southern Ethiopia), Dil Chora Referral Hospital (Eastern Ethiopia), Gondar University Hospital (Gondar), and Nazareth Hospital (Adama) (2,3,4,5,6,7,8). The studies consistently demonstrated that patients presenting with acute abdominal complaints were predominantly male (male to female ratios ranging from 4:1 to 2:1) and within 25 – 35 age group. Patients' most common symptoms, in order of frequency, were abdominal pain, vomiting, abdominal distension, and constipation while the major physical exam findings were abdominal tenderness, rebound tenderness, guarding, abdominal distension and abnormal bowel sounds (2–4). Intestinal obstruction was the leading cause of acute abdomen in all hospitals except the two urban locations, Nazareth Hospital and Tikur Anbessa Specialized Hospital. Hospital, which reported acute appendicitis as a major cause (2–8). This suggests that there is a shift in the primary cause of acute abdominal pain from intestinal obstruction to acute appendicitis in urban Ethiopia, which is consistent with the change in trend observed in other developing countries (9). In addition to providing the pattern of acute abdomen, these studies have demonstrated that acute abdomen is a major surgical problem in Ethiopia. In most of the aforementioned institutions, operations done for acute abdominal conditions accounted for roughly one-third of the total emergency operations. Most studies reported a post-operative complication rate of 30% and mortality rates ranging from 4% (at Mekelle Hospital) to 15.3% (at Tikur Anbessa Specialized Hospital) (2–4). The high prevalence of acute abdomen and its high post-operative complication and mortality rates are alarming and require further investigation.

There is a scarcity of published up-to-date information on the pattern of acute abdomen in Addis Ababa, Ethiopia. The only published study on acute abdomen in Addis Ababa was conducted at Tikur Anbessa Specialized Hospital 14 years ago (2). Most similar studies conducted in Ethiopia were done in a rural set-up and the majority of them in a public hospital setting. Here, we conducted a retrospective study of patients operated for acute abdomen at a private hospital in Addis Ababa, Ethiopia. The study was conducted with the aim of determining the pattern and outcome of surgically managed acute abdominal cases in an urban setup in general and in a private hospital setting in particular. We hoped this would provide us with the much needed upto-date information and a change in trend regarding etiologies, methods of diagnosis, modes of management, and outcomes of surgically managed acute abdomen.

## Materials and Methods

This was a retrospective cross-sectional study of acute abdominal cases operated between January 1, 2018 and August 1, 2019 at Teklehaimanot General Hospital. The hospital is a general hospital with 24 hours' emergency surgical service for acute abdominal and other surgical cases. The surgical division has a total of 5 general surgeons, 2 general practitioners, 3 anesthetists, 6 scrub nurses, 2 recovery unit nurses, and 8 surgical ward nurses.

Medical records of patients operated for non-traumatic acute abdominal complaints during the study period were collected and reviewed. Medical records of pediatric patients below the age of 13 and incomplete medical records were excluded from the study. Patients' medical records were reviewed to obtain data on patients' sociodemographic characteristics, clinical presentation, intraoperative finding, type of procedure performed, and postoperative course.

Data was analyzed using nonparametric statistical methods with the help of SPSS software package 26. Both descriptive and analytic statistics formed the mainstay of the statistical analysis. Using binary logistic regression, bivariate and multivariate analysis were performed. Odds ratio and P values were calculated to determine the significance of association between independent and dependent variables. Post-operative complication rate, length of post-operative hospital stay, and post-operative mortality were the dependent variables. A P value of less than 0.05 was considered to be significant. The study was done after obtaining permission from the hospital authorities and collected data was kept strictly confidential.

## Results

A total of 267 patients' medical records were reviewed. Of these, 162 patients were males accounting for 60.7% and 105 patients were females accounting for 39.3%. The male to female ratio was 1.5:1. The age of the patients ranged from 12 to 90 years and mean was 36 years±16 years (Table 1). Majority of patients came from Addis Ababa 210 (86.1%) and the rest were from outside of Addis Ababa. Seventy-eight (29.2%) of the patients were referrals from other facilities; a great proportion 74 (95%) of them from private clinics and the rest from government health centers 2 (2.5%) and government hospitals 2 (2.5%).

**Table 1:** Age and sex distribution of patients. Teklehaimanot General Hospital. Addis Ababa, Ethiopia. January 1, 2018 to August 1, 2019

Age (years)	Female (%)	Male (%)	Total (%)
13–20	24 (9)	20 (7.5)	44 (16.5)
21–30	28 (10.5)	53 (19.9)	81 (30.3)
31–40	20 (7.5)	40 (15)	60 (22.5%)
41–50	21 (7.9)	17 (6.4)	38 (14.2)
51–60	7 (2.6)	13 (4.9)	20 (7.5)
>60	5 (1.9)	19 (7.1)	24 (9)
Total	105 (39.3)	162 (61.7)	267 (100)

The average duration of symptoms before arrival was 71.1±84.4 hours (range 3 to 504 hours). Although there was no statistically significant association, duration of presentation was surprisingly shorter for referred patients by an average of 5.8 hours than non-referred ones (68.1±83.4 and 73.0±85.3 hours respectively). Only 85 (31.8%) of patients reached to the hospital within 24 hours or less of onset of symptoms. Equal proportion of patients 77 (28.8%) presented between 25 to 72 hours and the rest 105 (39.3%) presented after 72 hours of symptoms onset.

The major presenting clinical symptoms were abdominal pain in 241 (90.3%), vomiting in 163 (61.1%), and fever in 70 (26.2%). Abdominal direct tenderness 190 (71.1%) was the most frequent clinical sign. None of the patients had hypotension at presentation but tachycardia was seen in 57 (21.3%) patients. Table 2 summarizes the signs and symptoms of patients at presentation. Leukocytosis and nutrophilia were observed in 123 (50.4%) and 182 (70.3%) of the cases respectively. Leukocytosis was most commonly seen in patients with the diagnosis of generalized peritonitis 11 (84.8%), followed by acute appendicitis 101 (53.6%), small bowel obstruction 18 (51.2%), and large bowel obstruction 8 (47.4%). Abdominal ultrasound was requested in 228 (90.8%) patients, abdominal x-ray in 48 (18.9%), chest x-ray in 19 (7.1%), and abdominal CT-scan in 14 (5.5%) patients.

**Table 2:** Signs and symptoms of patients at presentation, Teklehaimanot General Hospital. Addis Ababa, Ethiopia. January 1, 2018 to August 1, 2019

Symptoms	Number	%	Signs	Number	percent
Abdominal pain	241	90.3	Direct tenderness	190	71.1
Vomiting	163	61.1	Rebound tenderness	61	22.8
Fever	70	26.2	Tachycardia	57	21.3
Failure to pass feces/flatus	45	16.8	Abdominal distention	35	13.1
Abdominal distention	31	11.6	Fever	29	10.9
Loss of appetite	25	9.4	Guarding	15	5.6
Diarrhea	16	5.9	Mass per abdomen	12	4.5
Dysuria	11	4.1	Hyperactive bowel sounds	13	4.9
Vaginal discharge	9	3.4	Positive signs of peritoneal fluid collection	11	4.1
Tenesmus	4	1.5	Other/associated signs	11	4.1
Other/associated symptoms	7	2.6			

Acute appendicitis was the most frequent cause of acute abdomen accounting for 193 (72.3%) of all cases of acute abdomen. The second most common cause was identified to be small bowel obstruction (SBO) 38 (14.3%), followed by large bowel obstruction (LBO) 19 (7.1%), and perforated peptic ulcer disease (PUD) 13 (4.9%). Major causes of SBO were post-op adhesion 14 (36.8%), hernia 13 (34.2%), intussusception 4 (12.2%), small bowel/ileocecal mass 4 (12.2%), and small bowel volvulus 3 (9.1%). Identified causes of LBO were sigmoid volvulus 11 (57.9%) and large bowel tumors 8 (42.1%). Results are shown in Table 3.

**Table 3:** Causes of acute abdomen. Teklehaimanot General Hospital. Addis Ababa, Ethiopia. January 1, 2018 to August 1, 2019

Cause	Number	Percent
Acute appendicitis	193	72.3
Small bowel obstruction	38	14.3
Post-op adhesion	14/38 (36.8%)	
Hernia	13/38 (34.2%)	
Intussusception	4/38 (12.2%)	
Small bowel/ileocecal mass	4/38 (12.2%)	
Small bowel volvulus	3/38 (9.1%)	
Large bowel obstruction	19	7.1
Sigmoid volvulus	11/19 (57.9%)	
Large bowel tumors	8/19 (42.1%)	
Perforated PUD	13	4.9
Ileo-sigmoid knotting	1	0.3
Pelvic inflammatory disease	1	0.3
Spontaneous primary peritonitis	1	0.3
Acute cholecystitis	1	0.3
Total	267	100

Average length of post-operative hospital stay was 3.3±2 days (range: 1 to 15 days). Patients who presented late to the hospital pre-operatively were found to have stayed longer in the hospital post-operatively (OR 1.59, 95% CI 1.03–2.45). Age of patients was also found to have a statistically significant positive correlation with the length of post-operative hospital stay (OR 1.25, 95% CI 1.00–1.57).

Post-operative complications were seen in 35 (14.8%) of the patients. Superficial surgical site infection, seen in nine (23.6%) patients, was the most frequently encountered complication. Pulmonary complications (four patients with hospital acquired pneumonia and one patient with pulmonary edema) were observed in five (14.3%) patients. Details are displayed in Table 4. All patients with superficial surgical site infections were managed with open wound care while all patients with post-operative abdominal collection and/or complete wound dehiscence were reoperated. Two out of the seven patients with post-operative SBO required re-operation.

**Table 4:** Post-operative complications. Teklehaimanot General Hospital. Addis Ababa, Ethiopia. January 1, 2018 to August 1, 2019

Complication	Number	percent
Superficial surgical site infection	9	23.6
Post-operative abdominal collection	8	22.8
Post-operative SBO	7	20
Pulmonary complications*	5	14.3
Complete wound dehiscence	2	5.6
Other complications*	4	11.4
Total	35	100

* Pulmonary complications: hospital acquired pneumonia and pulmonary edema

* Other complications: anastomotic leak, post-operative liver abscess, right ureteric injury, post-operative upper gastrointestinal bleeding

Great proportion 29 (82.8%) of the post-operative complications occurred in patients who presented ≥24 hours after onset of symptoms and a statistically significant association was found between duration of symptoms at presentation and occurrence of post-operative complications (OR 2.01, 95% CI 1.64–7.84). Age of patients was also found to have a statistically significant correlation with the occurrence of post-op complications with 22 (63.1%) complications occurring in patients above the age of 30 years (OR 1.51, 95% CI 1.89–3.59). In addition, patients who were tachycardic at presentation were found to be nearly 3 times more likely to develop post-operative complications (OR 2.85, 95% CI 1.03–6.82). Results are displayed in Table 5.

**Table 5:** Factors associated with occurrence of post-operative complication. Teklehaimanot General Hospital. Addis Ababa, Ethiopia. January 1, 2018 to August 1, 2019

	Post-operative complication		Bivariate		Multivariate	
	
	Yes	No	OR (lower, upper)	P value	OR (lower, upper)	P value
Age			2.02 (1.00, 1.04)	0.007	1.51 (1.89, 3.59)	0.02
13–20 years	2 (5.1%)	37 (94.9%)				
21–30 years	11 (15.4%)	66 (84.6%)				
31–40 years	6 (10.5%)	51 (89.5%)				
41–50 years	8 (25.7%)	26 (74.3%)				
51–60 years	2 (10.5%)	17 (89.5%)				
>60 years	6 (30.4%)	16 (69.6%)				
Sex			1.09 (0.53, 2.23)	0.80	0.95 (0.40, 2.24)	0.90
Male	22 (15.3%)	133(84.7%)				
Female	13 (14.1%)	85 (84.9%)				
Place of residence			1.10 (0.39, 3.11)	0.84	1.04 (0.32, 3.40)	0.94
Addis Ababa	30 (14.8%)	173(85.2%)				
Outside of Addis Ababa	5 (16.1%)	26 (83.9%)				
Presence of comorbidity			0.40 (0.16, 0.98)	0.46	0.64 (0.20, 2.05)	0.45
Yes	7 (27.6%)	21 (72.4%)				
No	28 (13.2%)	197(86.8%)				
Duration of presentation			3.36 (1.89, 2.08)	0.003	2.01 (1.67, 7.84)	0.01
≤24 hours	6 (9.6%)	75 (90.4%)				
25–72 hours	13 (17.1%)	63 (82.9%)				
>72 hours	16 (17.5%)	80 (82.5%)				
Fever			1.18 (0.24, 5.73)	0.83	1.29 (0.24, 6.95)	0.76
Yes	2 (18.2%)	9 (81.8%)				
No	31 (15.8%)	165(84.2%)				
Tachycardia			3.25 (1.43, 7.39)	0.005	2.85 (1.03, 6.82)	0.04
Yes	12 (32.4%)	25 (67.6%)				
No	22 (12.9%)	149(87.1%)				
Leukocytosis			0.89 (0.44, 1.80)	0.75	0.78 (0.33, 1.84)	0.57
Yes	18 (16.5%)	101(83.5%)				
No	17 (15%)	96 (85%)				

In this study, the overall mortality rate of surgically treated acute abdomen was 1.9%. The deaths occurred in patients with diagnoses of perforated PUD, LBO, strangulated inguinal hernia, and acute appendicitis with abdominal aortic aneurysm. Patients who presented late were more likely to die (OR 1.11, 95% CI 1.22–5.59) with four out of the five patients who died having a duration of symptoms at presentation of >72 hours. Post-operative mortality was slightly higher in female patients than males (OR 0.06, 95% CI 0.00–0.70). A statistically significant association was also found between age of patients and post-operative mortality (OR 3.95, 95% CI 1.39–11.96) (Table 6).

**Table 6:** Factors associated with post-operative mortality. Teklehaimanot General Hospital. Addis Ababa, Ethiopia. January 1, 2018 to August 1, 2019

	Post-operative mortality		Bivariate		Multivariate	
	
	Yes	No	OR(lower, upper)	P value	OR (lower, upper)	P value
Age			1.06 (1.01, 1.11)	0.01	3.95 (1.39, 11.96)	0.04
13–20 years	1 (2.6%)	38 (97.4%)				
21–30 years	0 (0%)	81 (100%)				
31–40 years	0 (0%)	59 (100%)				
41–50 years	1 (2.6%)	37 (97.4%)				
51–60 years	0 (0%)	19 (100%)				
>60 years	3 (12.5%)	21 (87.5%)				
Sex			0.15 (1.17, 1.41)	0.01	0.06 (0.00, 0.70)	0.03
Male	1 (0.6%)	160 (99.4%)				
Female	4 (3.8%)	100 (96.2%)				
Place of residence			4.29 (0.69, 26.6)	0.11	19.11 (0.79,	0.06
Addis Ababa	3 (1.4%)	206 (98.6%)			457.76)	
Outside of Addis Ababa	2 (5.9%)	32 (94.1%)				
Presence of comorbidity			0.50 (0.05, 4.64)	0.54	0.69 (0.04, 11.94)	0.80
Yes	1 (3.3%)	29 (96.7%)				
No	4 (1.7%)	231 (98.3%)				
Duration of presentation			2.44 (2.63, 7.44)	0.004	1.11 (1.22, 5.59)	0.03
≤24 hours	1 (1.2%)	82 (98.8%)				
25–72 hours	0 (0%)	77 (100%)				
>72 hours	4 (3.8%)	101 (96.2%)				
Fever			1.43 (1.78, 8.76)	0.71	0.34 (0.01, 0.78)	0.99
Yes	0 (0%)	12 (100%)				
No	5 (2.5%)	199 (97.5%)				
Tachycardia			1.18 (0.12, 10.8)	0.88	0.12 (0.004, 3.41)	0.21
Yes	1 (2.6%)	37 (97.4%)				
No	4 (2.2%)	175 (97.8%)				
Leukocytosis			0.24 (0.02, 2.25)	0.21	0.003 (0.001, 1.03)	0.06
Yes	4 (3.3%)	118 (96.7%)				
No	1 (0.8%)	119 (99.2%)				

## Discussion

There are few studies done on the general pattern of acute abdomen in Ethiopia but only one study from the capital, Addis Ababa (2–8). In our study, the male to female ratio was 1.5:1 and the majority of patients were in their 3rd to 4th decades of life, which was in agreement with previous studies done in the country and other African countries which consistently demonstrated that patients presenting with acute abdominal complaints were predominantly male (male to female ratios ranging from 4:1 to 2:1) and within 25 – 35 years' age group (2–9).

Studies from other centers in Ethiopia showed that patients' most common symptoms were abdominal pain, vomiting, abdominal distension and constipation while the major physical exam findings were direct abdominal tenderness, rebound tenderness, guarding, abdominal distension and abnormal bowel sounds (2–4). Similar findings were found in this study with most patients presenting with abdominal pain 241 (90.3%), vomiting 163 (61.1%), fever 70 (26.2%), abdominal direct tenderness 190 (71.1%), rebound tenderness 61 (22.8%), and tachycardia 57 (21.3%).

In our study, the average duration of symptoms before arrival was 71.1±84.4 hours (range 3 to 504 hours). Only 85 (31.8%) of patients reached to the hospital within 24 hours or less of onset of symptoms. This was lower than a report by Nega who found an average duration of symptoms before arrival of 88.4+/-87.9 hours (range 4 to 504 hours) (4). The fact that the majority 210 (86.1%) of the patients came from Addis Ababa might have contributed for this finding.

In earlier Ethiopian studies intestinal obstruction was reported to be the leading cause of acute abdominal pain (3–7). This was in contrary to our study which identified acute appendicitis as the most common cause of acute abdomen; observed in 193 (72.3%) of the cases. This was followed by SBO 38 (14.3%), LBO 19 (7.1%), and perforated PUD 13 (4.9%). The result was in agreement with two other studies from Ethiopia, Nazareth Hospital and Tikur Anbessa Specialized Hospital, which reported acute appendicitis as a major cause of acute abdomen (2,8). Studies from Nigeria (9) and Zaire (10) also depicted that acute appendicitis was the frequent cause of acute abdomen. This suggests that there is a shift in the primary cause of acute abdominal pain from intestinal obstruction to acute appendicitis in Ethiopia, which is consistent with the change in trend observed in other developing countries (9,10). This could possibly be due to the recent urbanization of most of these countries which led to a reduction in cases of small and large bowel volvulus (4,5). A study from Italy reported nonspecific abdominal pain as the leading cause of acute abdomen; accounting for 31.5% (16).

Adhesions were found to be the most frequent causes of small bowel obstruction in this study, which is in contrast to other studies that were done in central and southern Ethiopia and neighboring Kenya, where small bowel volvulus was reported to be the leading cause of small intestinal obstruction (5,6,7,12). But the results were in agreement to studies from certain urban areas in the country, such as Nazareth and Addis Ababa, and to a study from Nigeria by Adesunkanmi (2,8,11). Adesunkanmi has reported the changing pattern of intestinal obstruction from hernia, which has been the most frequent cause of small bowel obstruction in several African countries to a one dominated by adhesions (11). Similar results have been reflected in reports from developed nations (17,18). The widespread performance of therapeutic intra-abdominal surgery in the second half of the 20th century leading to an increase in the frequency of postoperative adhesion has been forwarded as an important reason for this shift in pattern ofetiologies for SBO. Our study showed that sigmoid volvulus accounted for 11 (57.9%) large bowel obstruction cases. Similar results were reported from Nazareth Hospital where sigmoid volvulus accounted for 60% of large bowel obstruction cases (13).

In this study, the mean length of post-operative hospital stay was 3.3±2 days (range: 1 to 15 days). This was lower than reports by Nega and Mc Conkey who reported mean length of hospital stay of 8.74 days and 13.5 days respectively (4,15). However, both of these studies analyzed the total duration of hospital stay, unlike ours where we considered only post-operative length of hospital stay. The overall early post-operative complication rate was found to be 14.8%; which was much lower than other studies (2,3,4,15). The overall mortality rate was 1.9%; which too was much lower than reports from Gondar (9.3%), Yirgalem (13.5%), Datubo (13.3%), and Tikur Anbessa Specialized Hospital (14.0%) (2,5,7,14). The low early post-operative complication rate and mortality rate reported in this series strongly correlates to the fact that majority of patients in this study were operated with the diagnosis of acute appendicitis, a surgical disease with a relatively low morbidity and mortality rates. In addition, the shorter duration of presentation and the fact that all patients in this series were operated by consultant surgeons might have contributed for this finding.

In our study, duration of presentation to the hospital was found to have a statistically significant positive correlation with length of post-operative hospital stay (OR 1.59, 95% CI 1.03–2.45), post-operative complication rate (OR 2.01, 95% CI 1.64–7.84), and overall mortality rate (OR 1.11, 95% CI 1.22–5.59). Several other studies have also attributed poor post-operative outcomes to patients' late presentation after onset of symptoms. Kotiso and Abdurahman reported that patients who went to Tikur Anbessa Specialized Hospital 2 days or less after onset of symptoms had a mortality rate of 7.6%, while those who presented greater than 2 days after onset of symptoms had a mortality rate of 25% (2). At Glenn Olson primary general hospital, Nega found that referred patients (who arrived 22.5 hours later than their non-referred counterparts) had longer hospital stays and more adverse outcomes (mainly wound infection and abscess collection).

The reasons for late presentation to the institution need to be studied further. However, Kotiso and Abdurahman and Nega have proposed the following reasons for late presentations: being referred to one or more institutions prior to arriving at surgical center (due to lack of surgical equipment or properly trained manpower at the original institution), failure to detect and refer patients early, poor transportation system and patients' lack of awareness of the acuteness of the problem (2,4).

Other than delay in presentation, post-operative complications (OR 1.51, 95% CI 1.89–3.59) and mortality (OR 3.95, 95% CI 1.39–11.96) were found to be higher in older patients with a statistically significant association. This could, predictably, be due to the frailty of these patients and the possible increment in physiologic decompensation in this age group. Similarly, tachycardia at the time of presentation was found to have a statistically significant association with occurrence of post-operative complications. As tachycardia could be a sign of sepsis in acute abdominal cases, it is not surprising to see it as a predictor of untoward outcomes after surgery (OR 2.85, 95% CI 1.03–6.82).

We believe this study will be of significant importance in providing basic information on the pattern as well as outcome of surgical management of acute abdomen in an urban setting. However, the utilization of secondary data sources in the background of poor data management system in the Hospital restricted the authors from including additional variables in the study to better describe the pattern and outcome of acute abdomen. Incomplete retrieval of medical records have led to a general reduction in the study sample and poor documentation of patient information on charts led to lack of uniformity and completeness of the medical records.

In conclusion, our study identified acute appendicitis as the predominant indication for acute abdomen operations, followed by small bowel obstruction secondary to adhesion. Length of post-operative hospital stay, early post-operative complication rate, and overall mortality rate were found to be significantly lower in our series than other reports. However, the relatively short duration of presentation, the predominance of acute appendicitis cases, and the fact that all patients in this series were operated by consultant surgeons might have contributed for this finding. However, it was found that delayed presentation to the Hospital resulted in higher morbidity and mortality of operated patients. Therefore, early detection and treatment of acute abdomen cannot be overemphasized. To this end, we recommend awareness creation for the general public and strengthening early detection and referral pathways for the health system. Findings from this study invite further multicenter studies with the inclusion of private and public institutions.
